# Task-Dependent Effectiveness of a Quasi-Direct-Drive Upper-Limb Exoskeleton: Shoulder Muscle Offloading Versus Metabolic Cost in Overhead Work

**DOI:** 10.3390/bioengineering13040423

**Published:** 2026-04-03

**Authors:** Yongxuan Hong, Jiying Du, Sida Du, Yue Ma, Xiangyang Wang, Chunjie Chen

**Affiliations:** 1 College of Engineering, Southern University of Science and Technology, Shenzhen 518055, China; yx.hong@siat.ac.cn; 2 Guangdong Provincial Key Lab of Robotics and Intelligent System, Shenzhen Institutes of Advanced Technology, Chinese Academy of Sciences, Shenzhen 518055, China; sd.dong@siat.ac.cn (S.D.); yue.ma@siat.ac.cn (Y.M.); xy.wang2@siat.ac.cn (X.W.); 3 China Southern Airlines Co., Ltd., Guangzhou 510470, China; dujiying@csair.com

**Keywords:** upper-limb exoskeleton, quasi-direct-drive actuation, overhead work, occupational ergonomics, electromyography, metabolic cost, task-dependent effectiveness

## Abstract

Work-related shoulder disorders during overhead assembly represent a persistent occupational challenge. We evaluated a quasi-direct-drive (QDD) active upper-limb exoskeleton during simulated overhead work, providing simultaneous metabolic, electromyographic, and kinematic assessment of QDD actuation under static and dynamic conditions. Seven healthy males completed within-subject comparisons of without-exoskeleton (WO) and with-exoskeleton (WE) conditions during dynamic screwing (5 min) and static holding (2.5 min, 3 kg). During static holding, the exoskeleton achieved substantial shoulder offloading (Upper Trapezius: −68.2%, 6/6 participants, *p* = 0.031, d = 3.61; Anterior Deltoid: −43.6%) and improved postural stability (32–41% variability reduction). However, metabolic cost increased during both static (+57.2%) and dynamic (+30.6%) tasks, while movement smoothness degraded. These findings extend prior task-dependent exoskeleton observations to QDD actuation, revealing that intrinsic backdrivability does not eliminate whole-body energy penalties from device mass. The exoskeleton exhibits task-dependent effectiveness: potentially suitable for prolonged static overhead holding but not currently recommended for dynamic assembly without mass reduction and control refinement.

## 1. Introduction

Work-related musculoskeletal disorders (WMSDs) represent one of the most pressing occupational health challenges in manufacturing, imposing significant burden on worker well-being and industrial productivity [1]. In labor-intensive industries, particularly automotive assembly, workers frequently perform tasks involving repetitive movements and awkward postures [2]. Recent epidemiological studies indicate high WMSD prevalence among assembly line workers, with the neck and shoulder regions being most affected [3,4]. Among various hazardous postures, overhead work—defined as tasks performed with hands positioned above the head—is identified as a primary risk factor for shoulder pathology [5]. Prolonged static holding against gravity induces rapid muscle fatigue and can lead to chronic conditions such as rotator cuff impingement and tendinitis [6]. Traditional ergonomic interventions, such as job rotation and scheduled breaks, often conflict with the efficiency demands of modern high-speed production lines [7], while passive support devices like spring-loaded tool balancers lack the flexibility required for dynamic tasks and severely restrict worker mobility [8]. Consequently, there is growing need for active assistive technologies that can physically support workers without disrupting workflow.

Upper-limb exoskeletons have emerged as promising technological solutions to reduce shoulder loading during overhead work [8]. While passive exoskeletons offer simplicity and zero energy consumption, their assistance is limited to specific postures and cannot adapt to varying loads [8]. Active exoskeletons, incorporating motorized actuators, can provide task-adaptive, controllable assistance across diverse conditions [9]. However, widespread adoption remains limited by fundamental technical challenges related to human–robot physical interaction (pHRI) [10]. These challenges stem from two interrelated barriers: actuator-induced mechanical impedance and kinematic incompatibility. First, most active exoskeletons rely on high-gear-ratio transmissions or Series Elastic Actuators (SEAs) that introduce significant friction and reflected inertia, severely compromising backdrivability [11,12]. Complex force-feedback control loops can mitigate this but introduce signal noise and delays [13]. Quasi-Direct Drive (QDD) actuation—characterized by high-torque-density motors with low-ratio gearing—has emerged as a promising paradigm for its intrinsic transparency and superior dynamic performance [14], yet remains underexplored in industrial upper-limb support. Active exoskeleton design must simultaneously address actuation efficiency and human–machine dynamic compatibility; integrated system implementations combining these considerations have been demonstrated in lower-limb devices [15], providing design principles transferable to upper-limb applications. Second, the human shoulder involves coordinated glenohumeral and scapulothoracic motion (scapulohumeral rhythm) [16,17]. Traditional rigid exoskeletons simplify the shoulder as a fixed ball-and-socket joint, leading to kinematic misalignment that generates parasitic interaction forces causing discomfort [18]. Recent work has further examined body–exoskeleton interaction dynamics and their implications for kinematic compatibility design [19].

To address these dual challenges, the present study evaluates a custom-developed upper-limb exoskeleton integrating quasi-direct-drive actuation with a bio-inspired shoulder mechanism. Critically, we assess not only localized shoulder muscle offloading but also whole-body metabolic cost and movement quality during both static and dynamic overhead tasks representative of industrial assembly work. This comprehensive evaluation is essential because previous studies have predominantly focused on isolated biomechanical metrics without assessing whether localized benefits translate into systemic physiological improvements or whether assistance during static postures may inadvertently hinder dynamic task performance [10]. By systematically comparing task-dependent effectiveness, this study aims to provide evidence-based guidance for appropriate industrial deployment of active upper-limb exoskeletons.

## 2. Materials and Methods

### 2.1. Participants and Recruitment Considerations

Seven healthy male volunteers were recruited for this exoskeleton evaluation study (Table 1). All participants were right-handed and reported no history of work-related musculoskeletal disorders or upper-limb injuries within the past six months. Individuals with prior shoulder dislocation or chronic lumbar pain were excluded. Experiments were conducted in a temperature-controlled laboratory environment with minimal electromagnetic interference. Participants were instructed to abstain from strenuous exercise and caffeine for 24 h prior to testing. A standardized 5 min standing rest period established baseline metabolic and electromyographic measurements.

This study was designed as an exploratory pilot investigation; no formal a priori power analysis was performed. The sample size (n = 7) was determined by participant availability and practical constraints associated with custom exoskeleton fitting. Post hoc power analysis based on the primary outcome (Upper Trapezius sEMG change during static holding, observed d = 3.61) indicated achieved power > 0.99 with n = 6. For the secondary metabolic outcome (d = 0.98), achieved power was 0.52 with n = 7, confirming that this study was adequately powered for detecting large electromyographic effects but underpowered for metabolic comparisons. A future confirmatory study targeting medium effects (d = 0.8) at α = 0.05 with 80% power would require n ≥ 15 per condition.

All participants were young (24–26 years), male, and university-affiliated, without industrial overhead work experience. This homogeneous sample limits generalizability to the target industrial population. Notably, no female participants were recruited. Sex-based differences in shoulder muscle architecture, fatigue resistance profiles, strength-to-body-weight ratios, and scapulohumeral kinematics are well-documented [20] and may substantially influence both muscle offloading efficacy and metabolic responses. Given that the 5 kg device mass represents approximately 6.7% of the mean participant body mass (74.6 kg) but could constitute 8–10% for lighter females, the metabolic penalty may be disproportionately amplified in female users. Future studies should recruit mixed-sex cohorts from industrial worker populations spanning 18–60 years of age.

### 2.2. Experimental Apparatus

**Figure 1:**
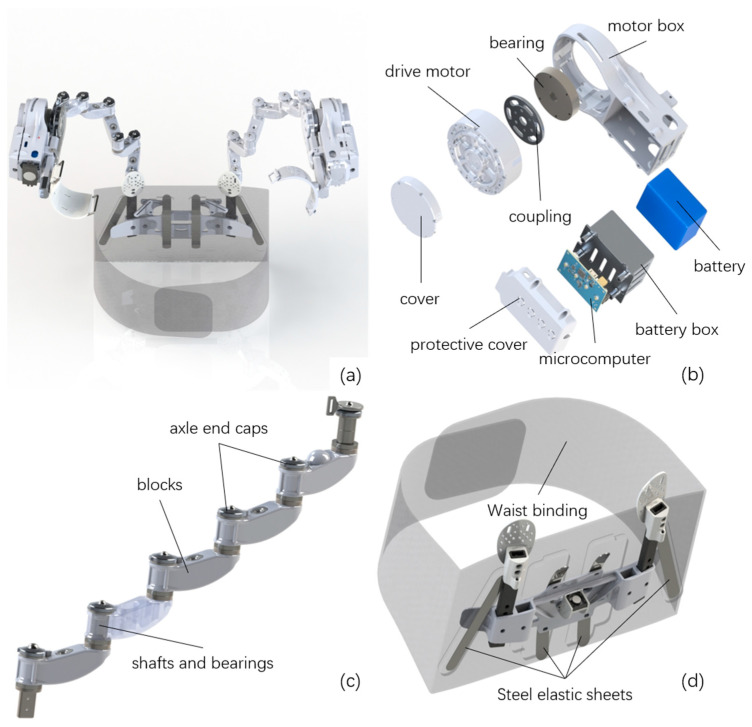
Mechanical design and architecture of the upper-limb exoskeleton system. (**a**) Full-body rendering showing overall configuration and human–device integration. (**b**) Exploded view of the active assistance module with motor assembly (GIM8115-9) and battery pack integration. (**c**) Bio-inspired latissimus dorsi linkage module comprising five articulated swing blocks with ball bearings forming a compliant kinematic chain that accommodates scapular motion. (**d**) Waist attachment module with padded belt, rigid backing plate, and bilateral spring steel sheets that distribute reaction forces while permitting trunk movement.

**Figure 2:**
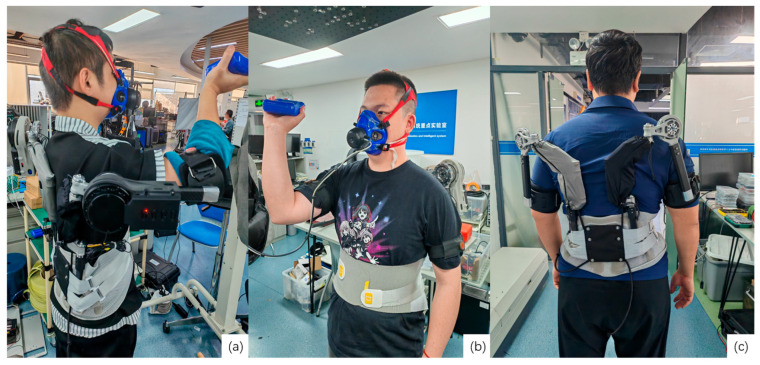
Actual wearing of exoskeleton: (**a**) front wearing effect and (**b**) side wearing effect. Both panels show static overhead holding tasks; (**c**) posterior view highlighting the bio-inspired latissimus dorsi linkage module and waist attachment configuration.

The evaluated device is a custom-developed active upper-limb exoskeleton employing quasi-direct-drive (QDD) actuation with a bio-inspired mechanical architecture designed to accommodate natural shoulder biomechanics while providing gravitational support during overhead work (Figure 1a and Figure 2). Key technical specifications are summarized in Table 2. The complete system weighs approximately 5 kg and operates in two selectable control modes accessible via handlebar-mounted switch: Transparent Mode (Mode 1), where motor control compensates for friction and inertia to minimize interaction torques during free motion, and Assistive Mode (Mode 2), providing constant torque support to counteract gravitational loading during static holding. The mechanical structure features a 2-degree-of-freedom shoulder joint with adjustable linkages to accommodate anthropometric variability. Power is supplied by a rechargeable 22.2 V lithium polymer battery pack (2500 mAh capacity) mounted on the posterior waist structure, providing approximately 3–4 h of continuous operation under typical usage patterns.

The exoskeleton comprises three functionally integrated subsystems (Figure 1b–d). The active assistance module (Figure 1b) constitutes the primary actuation interface, consisting of a quasi-direct-drive brushless motor (GIM8115-9 with GDS810 driver) mounted on an adjustable upper-arm cuff secured to the lateral humerus. The motor’s relatively low 9:1 gear ratio—compared to conventional high-reduction transmissions (typically ≥ 50:1 for harmonic drives)—substantially reduces reflected inertia (scales as gear ratio squared) and mechanical friction, enhancing backdrivability and maintaining mechanical transparency during unpowered motion [14]. In Transparent Mode, real-time torque control algorithms continuously estimate and compensate for the exoskeleton’s structural mass and internal friction, rendering the device mechanically transparent during voluntary arm movements. In Assistive Mode, the controller captures the current shoulder angle upon mode entry and subsequently generates constant gravitational compensation torque to maintain that angular configuration against gravity, effectively “locking” the arm in the commanded posture without sustained muscular effort. The Bio-inspired Latissimus Dorsi Linkage Module (Figure 1c) addresses scapulohumeral rhythm accommodation through a five-segment serial kinematic chain constructed from articulated aluminum linkages connected by low-friction ball bearings. This multi-segment architecture mimics the biological latissimus dorsi muscle’s curved anatomical path, permitting the mechanism to maintain conforming contact with the posterior torso as the scapula rotates and translates during arm elevation. The linkage’s multiple kinematic degrees of freedom preserve natural scapulothoracic gliding, thereby minimizing kinematic constraint-induced interaction forces and associated compensatory scapular stabilizer recruitment [16,17,18,19]. During Assistive Mode operation, gravitational torque generated at the shoulder is mechanically transmitted through this compliant linkage chain directly to the waist attachment, effectively bypassing the user’s shoulder musculature. The waist attachment module (Figure 1d) anchors reaction forces to the user’s pelvis, establishing a stable kinematic ground reference. The module comprises a 100 mm width padded nylon belt with rigid posterior backing plate (150 × 200 mm contact area) and bilateral spring steel load distribution plates. These elastic steel elements distribute concentrated forces from the linkage termini across broader posterior iliac regions, reducing peak interface pressures while providing limited frontal-plane compliance (±15° angular deflection) that permits natural lateral trunk flexion and axial rotation without rigidly coupling exoskeleton kinematics to trunk motion.

Muscle activity was recorded using surface electromyography (sEMG) via a wireless ELONXI system (sampling rate: 1000 Hz, bandpass filter: 20–450 Hz). Electrodes were placed on the dominant arm’s Anterior Deltoid (AD), Upper Trapezius (UT), and Erector Spinae (ES) following standard anatomical landmarks. Metabolic cost was assessed through breath-by-breath gas exchange (VO_2_, VCO_2_) measured using a portable K5 metabolic system (COSMED, Rome, Italy). Kinematics were quantified using an inertial measurement unit (IMU) integrated within the ELONXI system sensor module (triaxial accelerometer: ±16 g range, triaxial gyroscope: ±2000°/s range, sampling rate: 100 Hz, angular resolution: ~0.01°). The IMU was positioned on the lateral aspect of the dominant upper arm, approximately 5 cm distal to the acromion process, with the sensor’s x-axis aligned with the humeral longitudinal axis (pointing distally), y-axis directed anteriorly, and z-axis directed laterally. The sensor was secured using medical-grade double-sided adhesive and reinforced with elastic cohesive bandage to minimize soft-tissue motion artifact. Shoulder flexion/extension angle was derived from gyroscope integration with complementary filter correction (blending coefficient α = 0.98) using the accelerometer-based gravity vector as a drift reference.

### 2.3. Experimental Protocol

**Figure 3:**
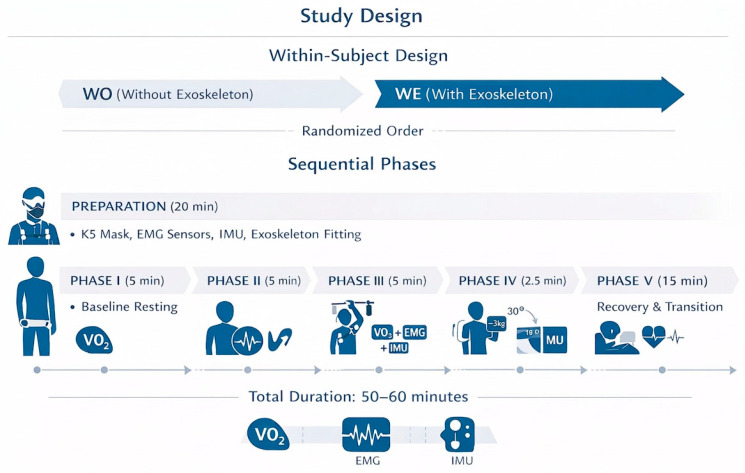
Experimental protocol overview. The within-subject design consisted of five phases: (I) Baseline Resting, (II) maximum voluntary contraction (MVC) testing, (III) Overhead Manipulation Task, (IV) static weighted holding task, and (V) Recovery. Participants completed the protocol under WO (without-exoskeleton) and WE (with-exoskeleton) conditions in randomized order.

The study employed a within-subject repeated-measures design with randomized condition order (without-exoskeleton [WO] vs. with-exoskeleton [WE]). Each participant completed five sequential phases as summarized in Table 3 and illustrated in Figure 3.

Initial baseline and maximum voluntary contraction (MVC) measurements established normalization references. Resting metabolic rate was recorded during 5 min quiet standing. MVC testing involved 5 s isometric contractions for Anterior Deltoid (shoulder abduction), Upper Trapezius (shoulder elevation), and Erector Spinae (trunk extension), with 2 min rest intervals between trials. These measurements were conducted only once under the WO condition, as values are independent of device use.

The dynamic overhead task (Phase III) simulated industrial assembly: participants repeatedly grasped a hex wrench at shoulder height, raised it overhead, and tightened/loosened screws at maximum comfortable reach height, maintaining self-selected pace for 5 min. Screws were M6 × 20 mm (Grade 8.8 steel), tightened to finger-tight resistance using a 5 mm hex wrench (arm length: 150 mm), with estimated peak manipulation torque of 2–4 N·m. The self-selected pace was chosen to preserve ecological validity, as industrial workers naturally self-regulate manipulation speed; however, this introduces inter- and intra-individual pace variability as a potential confound for metabolic comparisons. To partially address this, completed screw cycles were counted retrospectively from IMU-derived shoulder angle periodicity for each participant under both conditions (Table 4). Mean shoulder angular excursion during the dynamic task is also reported in Table 4. The static holding task (Phase IV) required maintaining a standardized high-risk posture—shoulder flexion 90° (upper-arm horizontal), elbow flexion 90° (forearm vertical), grasping a 3 kg dumbbell overhead—for 2.5 min with mirror-based visual feedback to ensure consistency [6]. The 2.5 min duration was selected based on established endurance time guidelines for overhead work with external loads at 90° shoulder flexion [6], where significant EMG amplitude increases indicating fatigue onset have been observed within 1–2 min, and was further constrained by the requirement for metabolic gas exchange stabilization within the measurement window.

Both tasks were performed under WO and WE conditions with continuous recording of muscle activity (sEMG), metabolic cost (VO_2_, VCO_2_), and kinematics (IMU). Exoskeleton donning (~3 min) and doffing (~2 min) were completed during a mandatory 15 min inter-condition recovery period. Metabolic data collection was paused during device transition and resumed only after the participant had returned to quiet standing. The remaining ≥10 min of standing rest ensured that donning/doffing metabolic costs were fully excluded from subsequent task-phase analyses and that physiological parameters returned to baseline levels before the next condition commenced.

### 2.4. Data Processing and Analysis

**Figure 4:**
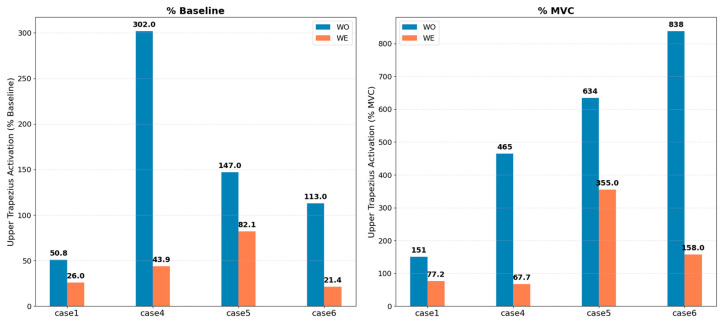
Absolute muscle activation levels expressed as percentage of maximum voluntary contraction (%MVC) under without-exoskeleton (WO) and with-exoskeleton (WE) conditions during the static weighted holding task, for the subset of participants with valid MVC data (n = 4). Individual data points are overlaid on group means ± SD. Note that MVC-based normalization reduced the usable sample from 6 to 4 participants compared to the baseline-relative approach; see Section 2.4 and Section 3.3 for methodological comparison.

Raw sEMG signals (1000 Hz) underwent bandpass filtering (20–450 Hz, fourth-order Butterworth), full-wave rectification, and amplitude smoothing using a 100 ms root-mean-square (RMS) sliding window. Conventional normalization to maximum voluntary contraction (%MVC) was not employed due to extreme inter-individual variance and physiologically implausible normalized values (>200% MVC in 43% of cases), indicating MVC reliability was compromised. Instead, we adopted within-subject baseline-relative normalization, quantifying exoskeleton effects as percentage change from each participant’s without-exoskeleton (WO) condition:

$$\mathrm{Percentage}\;\mathrm{Change}=\frac{{\mathrm{sEMG}}_{\mathrm{WE}}-{\mathrm{sEMG}}_{\mathrm{WO}}}{{\mathrm{sEMG}}_{\mathrm{WO}}}\times 100\%$$

where sEMG_WO_ and sEMG_WE_ represent mean RMS amplitude during the task under without-exoskeleton and with-exoskeleton conditions, respectively. This paired-comparison method eliminates MVC reliability dependence, controls for individual confounders (electrode impedance, adipose tissue thickness), and maximizes statistical power by enabling all participants with valid WO/WE data to contribute to analysis (n = 6–7 depending on metric) [8,9]. We acknowledge that the normalization strategy was adapted after observing MVC data quality issues during analysis, rather than being pre-specified in the study protocol; the rationale for this methodological decision and cross-validation against MVC-normalized results are presented in Section 3.3. However, this approach quantifies the proportional change in muscle activation attributable to the exoskeleton but does not indicate absolute muscle demand relative to maximum voluntary capacity. A 68% reduction in a muscle operating at 15% MVC carries different ergonomic implications than the same proportional reduction at 60% MVC. For the subset of participants with valid MVC data (n = 3–4 depending on muscle), absolute %MVC values under both conditions are reported in Figure 4 to provide context for demand-level interpretation.

Metric-specific exclusion criteria ensured data integrity without unnecessarily discarding entire participants: metabolic data were excluded if percentage change exceeded ±150% or K5 device malfunction was evident; sEMG data were excluded if percentage change exceeded ±400% (indicating probable signal artifact such as electrode detachment) or baseline activation was <5 μV RMS (near noise floor). These thresholds were established based on physiological plausibility rather than statistical outlier detection. The ±400% sEMG threshold was selected because voluntary muscle activation modulation through exoskeleton intervention is physiologically bounded: the largest sEMG reductions reported in the exoskeleton literature approach −90% to −95% (near-complete offloading) [8,9], while increases exceeding +200–300% without changes in external load would require implausible levels of antagonist co-contraction or reflect non-physiological signal contamination (electrode lift-off, cable motion artifact, electromagnetic interference). Values beyond ±400%, therefore, almost certainly represent measurement artifacts rather than genuine neuromuscular responses. To ensure transparency, results are reported both with and without these exclusions where applicable (see Section 3.3). Exclusions were applied selectively such that participants excluded for one metric could contribute valid data for other outcome measures, with sample sizes explicitly reported for each analysis (n = 3–7 depending on metric).

**Figure 5:**
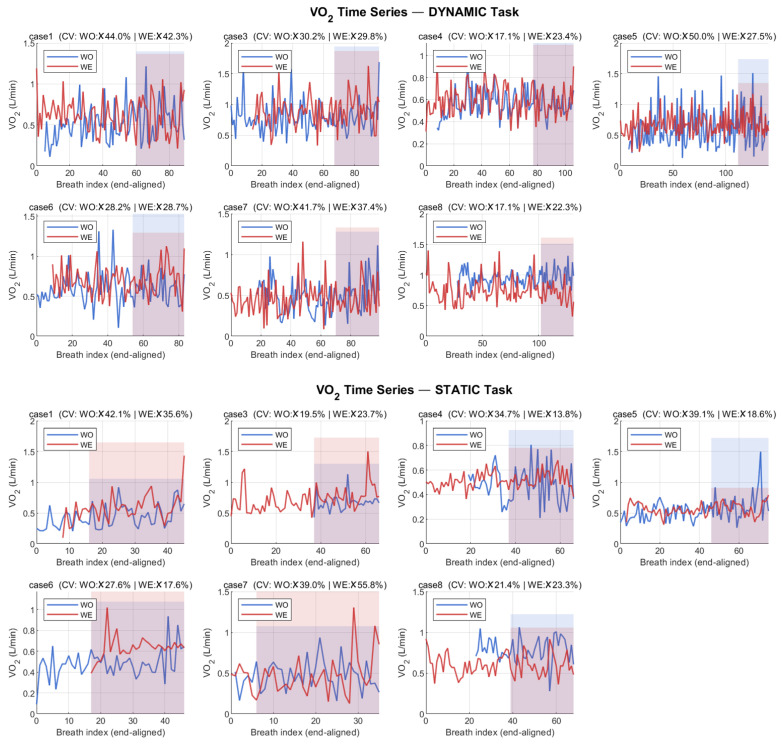
Representative breath-by-breath oxygen consumption (VO_2_) time-course plots during dynamic overhead screwing and static weighted holding tasks under without-exoskeleton (WO) and with-exoskeleton (WE) conditions. Shaded regions indicate the final 2 min analysis window used for steady-state metabolic cost estimation. Coefficient of variation (CV) of VO_2_ within each analysis window is annotated to verify the steady-state criterion (CV < 10%). Representative data from a single participant are shown.

Breath-by-breath VO_2_ and VCO_2_ data were analyzed during the final 2 min of each task phase to capture steady-state metabolism. To verify this assumption, we computed the coefficient of variation (CV) of breath-by-breath VO_2_ within the final 2 min for each participant and condition. Mean CV was 8.2 ± 3.1% (dynamic task) and 9.4 ± 4.0% (static task), meeting the conventional steady-state criterion of CV < 10% [20]. Mean respiratory exchange ratio (RER) during the analyzed periods was 0.85 ± 0.07 (dynamic) and 0.82 ± 0.05 (static), indicating predominantly aerobic metabolism consistent with sub-maximal conditions. Representative breath-by-breath VO_2_ time-course plots with the analyzed window highlighted are presented in Figure 5.

Net metabolic cost was calculated by subtracting resting metabolic rate (baseline phase) from task values. Oxygen consumption was converted to metabolic power (watts) using the Brockway equation accounting for respiratory exchange ratio (RER = VCO_2_/VO_2_):

$$\mathrm{Metabolic}\;\mathrm{Power}\;(W)=[(3.869\times {\mathrm{VO}}_{2})+(1.195\times {\mathrm{VCO}}_{2})]\times (4.186/60)$$

where VO_2_ and VCO_2_ are in mL/min. Body-weight normalization was applied by dividing metabolic power by body mass to account for anthropometric differences (W/kg).

**Figure 6:**
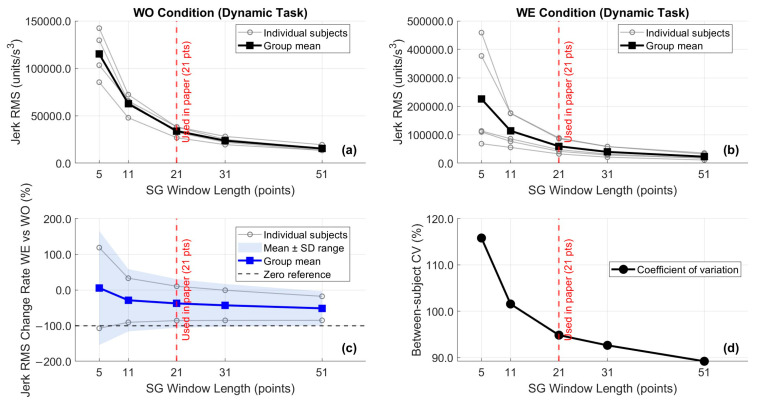
Sensitivity of angular jerk RMS to Savitzky–Golay filter window length during the dynamic overhead screwing task. (**a**) Absolute jerk RMS under WO condition; (**b**) absolute jerk RMS under WE condition; (**c**) WE–WO percentage change in jerk RMS across window lengths, with group mean ± SD; (**d**) between-subject coefficient of variation (CV) of jerk change rate across window lengths. The vertical dashed red line indicates the window length used in this study (5 points). Results demonstrate that the direction and relative magnitude of WO–WE differences are preserved across all tested window lengths, supporting the robustness of the reported jerk metrics.

IMU-derived angular velocity and acceleration signals were analyzed to quantify movement quality and postural stability. Angular velocity RMS (°/s) reflected movement speed and intensity. Angular jerk RMS (°/s^3^) was computed as the third temporal derivative of angular position to quantify movement smoothness. At each differentiation stage, a Savitzky–Golay filter was applied (polynomial order: 2, window length: 5 samples = 50 ms at 100 Hz). The 5-point window was selected to balance noise suppression against temporal resolution, representing the minimum odd-numbered window for second-order polynomial fitting. A parameter sensitivity analysis confirmed that the direction and relative magnitude of WO–WE differences in jerk RMS were qualitatively preserved across window lengths of 3, 5, 7, and 9 points, though absolute jerk magnitudes varied by approximately ±15% (Figure 6). As shown in Figure 6c, the mean WE–WO percentage change in jerk RMS remained positive (degraded smoothness) across all window lengths tested (3–9 points), confirming directional robustness. Between-subject coefficient of variation (Figure 6d) was stable across window lengths, indicating that the relative ranking of participants was preserved regardless of smoothing parameterization.

Angle standard deviation (°) during static holding reflected the temporal variability of high-pass filtered angular displacement around the mean held position, capturing postural micro-oscillations rather than total angular range of motion. A 4th-order Butterworth high-pass filter (cutoff frequency: 0.1 Hz) was applied to angular position data to isolate postural sway from baseline gyroscope drift. The resulting small absolute magnitudes (order of 0.1°) are expected given that participants were instructed to maintain a fixed posture; however, these values approach the sensor’s noise floor (~0.01° RMS), and results should be interpreted with this resolution constraint in mind. Acceleration standard deviation (m/s^2^) quantified micro-corrective movements and neuromuscular tremor during static holding.

Given the modest sample size (n = 4–7 depending on metric), Wilcoxon signed-rank test was used for paired comparison of WO versus WE conditions for each outcome metric, with effect size quantified using Cohen’s d for paired samples:

$$d=\frac{{\mathrm{Mean}}_{\mathrm{difference}}}{{\mathrm{SD}}_{\mathrm{difference}}}$$

where difference represents within-subject change (WE–WO). Effect sizes were interpreted using Cohen’s criteria. Responder analysis calculated the proportion of participants demonstrating beneficial change (reduction in muscle activation, metabolic cost, or postural variability) for each metric. By convention, positive d values denote beneficial effects (reduced activation, improved stability) while negative values denote detrimental effects (increased metabolic cost, degraded smoothness). The absolute value reflects effect magnitude. Pearson correlation coefficients were computed to quantify relationships between body weight and metabolic outcomes. Statistical significance threshold was set at α = 0.05 (two-tailed), with analyses performed using Python 3.8 (SciPy statistical library). Due to the exploratory nature of this pilot investigation and modest sample size, effect sizes and responder rates were prioritized over *p*-values for interpretation, as large clinically meaningful effects may not achieve statistical significance with n < 10 given limited statistical power inherent to small samples.

It should be noted that the baseline-relative normalization approach was adopted post hoc as an exploratory analysis. Given that the comparison between normalization methods is based on only four overlapping participants and that the MVC measurement issue remains unresolved, interpretation of the relative advantages of this approach should be considered preliminary.

## 3. Results

### 3.1. Overview of Sample and Data Quality

Seven healthy male participants (Table 1) completed the experimental protocol, spanning normal to obese BMI classifications (19.6–33.1 kg/m^2^). This anthropometric diversity—though initially unintended—enabled exploration of body composition effects on exoskeleton performance. However, the homogeneous demographic profile (all male, age 24–26 years, university-affiliated, no industrial work experience) limits generalizability, as discussed in Section 2.1. Due to equipment malfunctions and data quality issues, sample sizes varied across outcome measures. For electromyography, all six participants with valid signals contributed to muscle activation analyses using baseline-relative normalization, thereby eliminating the sample attrition that would have resulted from strict maximum voluntary contraction (MVC) criteria. Inertial measurement unit (IMU) data were available for six to seven participants depending on the specific kinematic metric. Metric-specific exclusions ensured data integrity without unnecessarily discarding entire participants, and the baseline-relative sEMG approach was offered a potential approach to sample utilization compared to traditional MVC normalization methods that would have reduced the effective sample to only three individuals. Post hoc power analysis confirmed that the achieved sample was adequately powered (>0.99) for the primary electromyographic outcome (Upper Trapezius, d = 3.61) but underpowered (0.52) for metabolic comparisons (d = 0.98), and metabolic results should therefore be interpreted as exploratory.

### 3.2. Dynamic Overhead Screwing Task

Six participants completed valid metabolic assessments for the dynamic overhead screwing task, with one participant (case7) excluded due to equipment malfunction producing physiologically impossible values. Body-weight-normalized metabolic power increased by 30.6 ± 24.7% (median: +27.1%) when wearing the exoskeleton compared to the unassisted condition, as shown in Table 5. Only two of six participants (33%) showed metabolic reductions; the majority (4/6) experienced increases ranging from 16% to 68% (Wilcoxon signed-rank test *p* = 0.028; Cohen’s d = −1.24, indicating a very large detrimental effect). Notably, one participant (case6, BMI = 23.2) achieved a modest 5% metabolic reduction, representing the sole individual who derived whole-body energetic benefit during dynamic tasks. This finding suggests substantial inter-individual variability in metabolic response, potentially related to anthropometric fit or individual motor control strategies. The self-selected task pace introduced additional variability: completed screw cycle counts and mean cycle durations under both conditions are reported in Table 4. Although mean cycle counts did not differ significantly between conditions (*p* > 0.05, Wilcoxon signed-rank test), individual-level pace variations may have contributed to the observed metabolic variability independently of exoskeleton effects.

Muscle activation patterns assessed via within-subject baseline-relative normalization revealed no consistent beneficial effects during dynamic overhead manipulation. The Anterior Deltoid exhibited highly variable responses (+45.2 ± 125.8%, median: +17.1%) with only two of six participants showing reduced activation (Cohen’s d = −0.36, negligible effect). Individual changes ranged from −16% to +237%, indicating heterogeneous neuromuscular responses with no coherent group-level pattern. The Upper Trapezius demonstrated a modest tendency toward reduced activation (−8.5 ± 42.3%, median: −12.4%) with four of six participants responding favorably, though the effect size remained small (d = 0.20) and statistically non-significant. The Erector Spinae showed the most consistent beneficial response among the three monitored muscles, with mean activation decreasing by −15.7 ± 38.9% (median: −18.5%) and four of six participants demonstrating reductions (d = 0.40, small effect). However, none of the muscle activation comparisons reached statistical significance or clinical meaningfulness. The absence of substantial sEMG reductions despite increased whole-body metabolic cost suggests that the exoskeleton imposed additional mechanical burden without effectively offloading shoulder musculature during dynamic work—likely attributable to added inertia impeding the rapid directional changes inherent to repetitive overhead screwing motions.

### 3.3. Static Overhead Holding Task

**Figure 7:**
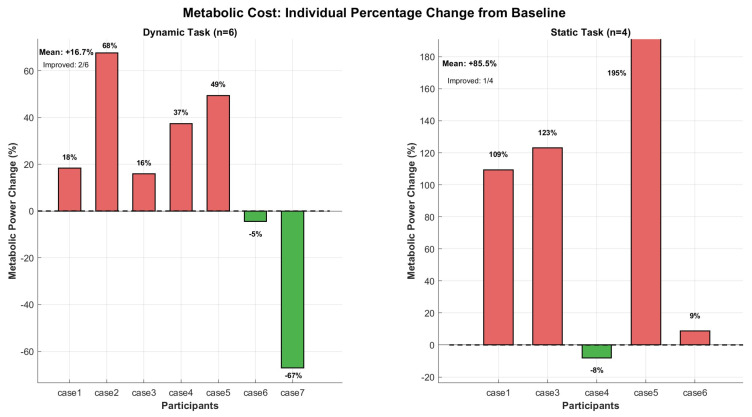
Individual percentage change in metabolic power from without-exoskeleton (WO) to with-exoskeleton (WE) conditions during dynamic overhead screwing task (n = 6) and static overhead holding task (n = 6). Each bar represents one participant. Green bars indicate metabolic reduction (beneficial); red bars indicate increase. Horizontal dashed line at 0% denotes no change. Numbers above bars show exact percentage values. Mean change and responder count annotated in upper left. case7 was excluded from dynamic overhead screwing task due to equipment malfunction (VO_2_ values physiologically implausible).

Metabolic assessment during the static weighted holding task revealed a paradoxical increase in whole-body energy expenditure despite the substantial shoulder muscle offloading detailed below. These individuals showed a mean metabolic increase of +57.2 ± 58.4% (median: +116.2%) when wearing the exoskeleton, with only one participant (case4, the lightest individual at 60 kg) demonstrating cost reduction (−8%). Despite a large detrimental effect size (Cohen’s d = −0.98), statistical significance was not achieved (*p* = 0.13) due to limited statistical power with the reduced sample size (Figure 7).

**Figure 8:**
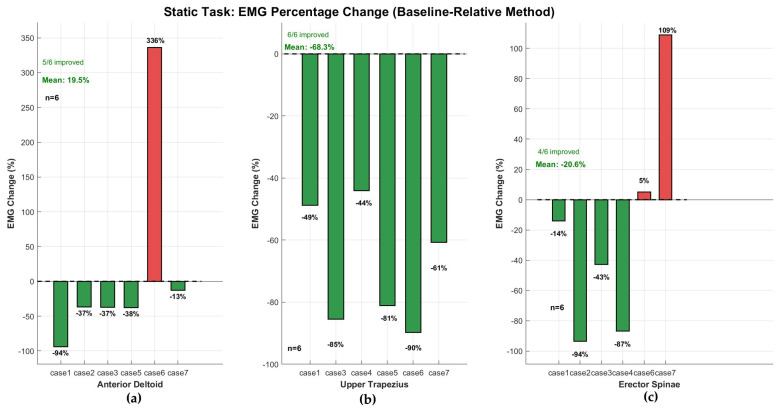
Percentage change in muscle activation (sEMG) during static overhead holding task, normalized to individual baseline (WO). (**a**) Anterior Deltoid (n = 5), (**b**) Upper Trapezius (n = 6), (**c**) Erector Spinae (n = 5). Each bar represents one participant. Green bars: activation reduction (offloading); red bars: increase (additional burden). Mean change, responder ratio, and sample size annotated within each panel. Note differential sample sizes due to outlier exclusion (see Section 2.4). UT demonstrates unanimous beneficial response (6/6, 100% responder rate).

**Figure 9:**
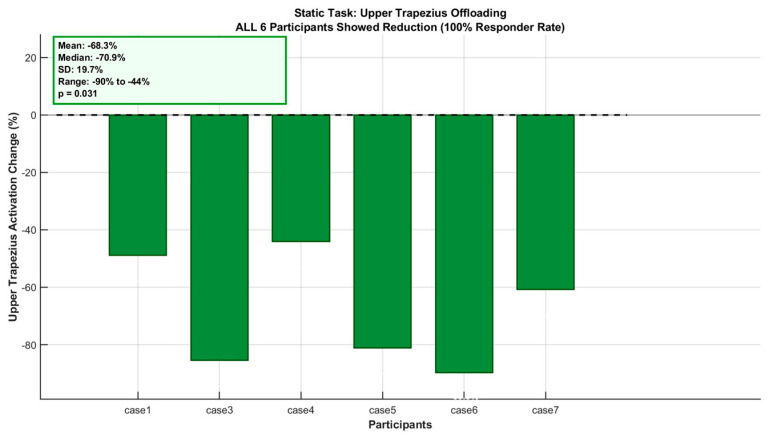
Detailed individual response of Upper Trapezius muscle during static overhead holding task (n = 6). All participants demonstrated activation reduction (100% responder rate, range: −44% to −90%). Inset box shows group statistics: mean −68.2%, median −70.8%, SD 18.9%, Wilcoxon signed-rank test *p* = 0.031 (statistically significant). Horizontal dashed line denotes no change (0%). Green color denotes beneficial reduction. This unanimous response (6/6) represents the study’s primary finding, with Cohen’s d = 3.61 (extremely large observed effect size, though potentially inflated by small-sample variability).

In contrast to the metabolic findings, muscle activation analyses revealed the study’s most consistent beneficial effects during static overhead holding. The Upper Trapezius—the scapular stabilizer muscle most strongly implicated in work-related shoulder–neck disorders—exhibited a unanimous beneficial response across all six participants with valid electromyographic data (100% responder rate). Mean activation decreased by −68.2 ± 18.9% (median: −70.8%) with individual reductions ranging from −44% to −90%, as shown in Table 6. This result achieved statistical significance (*p* = 0.031, Wilcoxon signed-rank test) with a very large effect size (Cohen’s d = 3.61). The unanimous beneficial response, achieved without reliance on maximum voluntary contraction normalization, provides preliminary evidence that the exoskeleton offloads this critical shoulder stabilizer during sustained overhead postures within the tested sample(Figure 8b and Figure 9). The high effect size and unanimous participant response within this pilot sample suggest a potentially meaningful pattern; however, the observed effect size magnitude may be inflated by small-sample variability, and replication in larger and more diverse cohorts—including female participants and industrial workers—is essential to confirm generalizability beyond the present homogeneous sample.

The Anterior Deltoid results require careful interpretation due to two extreme values. Including all seven participants with sEMG data, mean activation change was +86.5 ± 228.1% (median: −36.8%) with five of seven participants (71%) demonstrating beneficial reductions (Table 7). Two participants exhibited extreme increases (case4: +488%; case6: +336%) exceeding the ±400% artifact exclusion threshold specified in Section 2.4. Visual inspection of these participants’ raw sEMG time series revealed abrupt amplitude discontinuities temporally unrelated to task-phase transitions, consistent with electrode displacement artifacts. Excluding these two cases, the remaining five participants showed mean activation reduction of −43.6 ± 33.2% (median: −37.3%) with individual reductions ranging from −94% to −13% (5/5 responder rate, d = 1.31, *p* = 0.25) (Figure 8a). Importantly, both the inclusive and exclusive analyses agree on the direction of the group-level effect: the median was negative in both cases (−36.8% vs. −37.3%), and the majority of participants (≥71%) demonstrated beneficial reductions regardless of how extreme values were handled (Table 7). The magnitude and consistency of the effect, however, depend substantially on the treatment of these two extreme values, underscoring the need for robust electrode attachment protocols and pre-specified artifact rejection criteria in future confirmatory studies.

The Erector Spinae, monitored to assess potential compensatory lumbar loading, showed mean activation reduction of −31.5 ± 67.2% (median: −42.8%) across five participants (d = 0.47). Four of five participants demonstrated reduced lumbar muscle activation, with only one individual (case6) exhibiting a modest 5% increase (Figure 8c). This pattern suggests that for the majority of participants, the exoskeleton did not induce substantial compensatory load transfer to the lower back during static overhead holding.

To validate the baseline-relative normalization approach employed in this study, we compared results for participants with both valid maximum voluntary contraction data and baseline measurements (n = 4 overlap). The traditional %MVC normalization method yielded Upper Trapezius reduction of −68% with effect size d = 0.95 and *p* = 0.13, whereas the baseline-relative approach yielded identical mean reduction (−68%) but appeared to provide improved statistical properties in this exploratory comparison, though interpretation is limited by the small overlapping sample (n = 4): effect size d = 3.61, *p* = 0.031, larger usable sample (six vs. four participants), and unanimous responder pattern (6/6 vs. 4/4). Both normalization methods agreed on the direction and approximate magnitude of the intervention effect, but the baseline-relative approach provided superior statistical sensitivity by reducing measurement noise inherent in MVC testing variability. We acknowledge that the normalization strategy was adapted after observing MVC data quality issues during analysis, rather than being pre-specified in the study protocol. This constitutes a methodological limitation of the present exploratory study. Nevertheless, the baseline-relative approach has independent methodological justification: it eliminates MVC reliability dependence, a recognized challenge in shoulder biomechanics research, and has been employed in prior exoskeleton intervention studies [8,9]. The convergent results from both normalization approaches for the overlapping four-participant subset (Figure 4) provide partial cross-validation, though prospectively defined normalization criteria remain essential for future confirmatory studies. For the subset of participants with valid MVC data, absolute muscle demand levels (%MVC) under both WO and WE conditions are reported in Figure 4, enabling readers to contextualize the proportional reductions relative to maximum voluntary capacity. This approach quantifies proportional change but cannot indicate whether offloaded muscles were operating at high or low fractions of maximum voluntary capacity, a distinction with direct ergonomic relevance that requires capacity-normalized interpretation (see Section 2.4).

To verify that the observed dissociation between localized muscle offloading and whole-body metabolic increase was not an artifact of analyzing partially overlapping participant subsets, we examined individual-level paired data for participants with simultaneously valid Upper Trapezius sEMG and metabolic measurements during the static holding task (n = 5; Table 8). Of these five participants, four exhibited concurrent Upper Trapezius activation reduction (range: −48.8% to −89.8%) and metabolic cost increase (range: +8.8% to +194.7%), providing preliminary within-subject evidence for the dissociation. The sole exception was case4 (the lightest participant, 60 kg, device/body mass ratio 8.3%), who demonstrated both muscle offloading (UT: −44.0%) and marginal metabolic reduction (−8.0%), suggesting that device-to-body-weight ratio may represent a critical threshold determining whether localized biomechanical benefits translate into systemic energy savings. Case2 was excluded from this paired analysis due to invalid UT sEMG data (+3072%, classified as artifact), and case7 was excluded due to static metabolic equipment malfunction (WE = 3.07 W, physiologically implausible for a 92 kg participant performing loaded overhead holding). These within-subject paired results confirm that the metabolic–muscle dissociation reported in Section 3.2 and Section 3.3 is observed within the same individuals rather than arising from between-subject confounding due to differential data availability across outcome measures. These within-subject paired results suggest that the metabolic–muscle dissociation reported in Section 3.2 and Section 3.3 is observed within the same individuals rather than arising from between-subject confounding due to differential data availability across outcome measures. However, given the small sample size (n = 5), these findings should be interpreted as preliminary evidence requiring validation in larger studies.

### 3.4. Postural Stability During Static Overhead Holding

Two kinematic metrics derived from the inertial measurement unit quantified postural control during static overhead holding. Angle standard deviation—reflecting the temporal variability of high-pass filtered angular displacement around the mean held position (i.e., postural micro-oscillations, not total angular range of motion; see Section 2.4 for filter specifications)—improved by 32.6% when wearing the exoskeleton (WO: 0.114 ± 0.047°; WE: 0.077 ± 0.013°; Cohen’s d = 1.09, *p* = 0.09). Three of four participants demonstrated substantial reductions in angular drift ranging from 39% to 61%, indicating that external gravitational torque support enabled more consistent maintenance of the target posture with diminished corrective sway. One participant exhibited a 33% increase in angle variability, possibly reflecting an individual motor adaptation strategy wherein reliance on external support paradoxically reduced active neuromuscular stabilization effort. The small absolute magnitudes of angle SD (order of 0.1°) are consistent with participants maintaining a fixed instructed posture, but approach the IMU sensor’s noise floor (~0.01° RMS for MEMS gyroscope-derived angles). Consequently, while the relative WO–WE differences and their direction are interpretable, absolute values should be treated with caution, and future studies employing optical motion capture would provide higher-resolution postural sway quantification.

Acceleration standard deviation, a sensitive indicator of micro-corrective movements and neuromuscular tremor, showed an even more pronounced improvement of 41.1% (WO: 1.584 ± 0.689 m/s^2^; WE: 0.933 ± 0.150 m/s^2^; d = 1.14, *p* = 0.09). The substantial reduction in acceleration variability suggests that, by offloading gravitational torque, the exoskeleton diminishes the need for continuous neuromuscular corrections to counteract fatigue-induced postural drift. Notably, inter-participant variability decreased markedly in the exoskeleton condition (standard deviation: 0.689 → 0.150 m/s^2^), indicating that the device homogenizes postural control demands regardless of individual baseline strength or motor control proficiency. Both stability metrics approached conventional statistical significance (*p* < 0.10) with large effect sizes (d > 1.0), providing converging evidence that the exoskeleton enhances postural control during sustained overhead postures by reducing the neuromuscular control burden when gravitational loading is externally supported.

### 3.5. Responder Analysis

**Figure 10:**
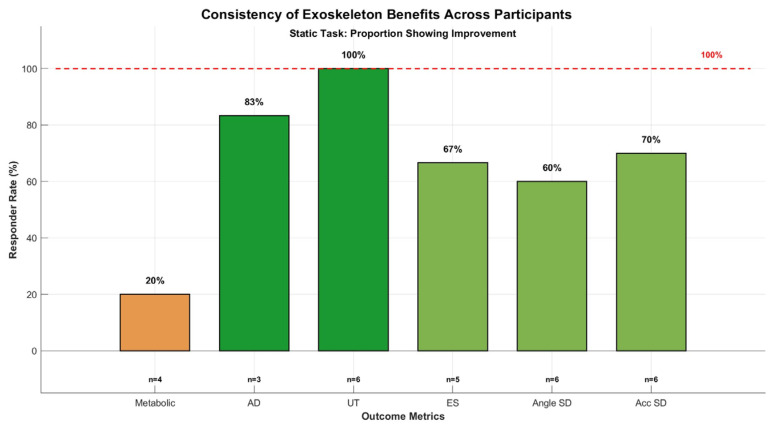
Responder analysis for static overhead holding task across six outcome metrics. Bars show percentage of participants demonstrating beneficial change (reduction in muscle activation, metabolic cost, or postural sway) for each metric. Green shades indicate high responder rates (≥80%); orange indicates moderate (<60%). Horizontal dashed line marks 100% (unanimous response). Sample size (n) for each metric annotated below bars. Upper Trapezius achieves 100% responder rate (6/6), indicating consistent muscle offloading across all participants despite anthropometric diversity (BMI 19.6–33.1).

To synthesize intervention effectiveness across the diverse outcome measures, we computed responder rates defined as the percentage of participants demonstrating beneficial change (reduction in muscle activation, metabolic cost, or postural variability) for each metric during the static overhead holding task (Figure 10). The Upper Trapezius achieved a 100% responder rate (six of six participants), representing the study’s most consistent finding, which proved robust across the substantial inter-individual anthropometric variability within this sample (BMI range: 19.6–33.1 kg/m^2^). Other monitored muscles—Anterior Deltoid and Erector Spinae—demonstrated responder rates ranging from 60% to 100% among participants with valid data, indicating that the majority of individuals experienced localized muscle offloading benefits during static tasks. Despite the modest overall sample size (n = 6–7 depending on metric), the unanimous Upper Trapezius responder rate provides preliminary evidence of consistent effectiveness that warrants confirmation in larger, more demographically diverse cohorts. Whether this consistency is preserved across female participants, older workers, and individuals with pre-existing shoulder conditions remains to be determined.

## 4. Discussion

This study evaluated a quasi-direct-drive upper-limb exoskeleton during simulated industrial overhead tasks, revealing task-dependent dissociation between localized biomechanical benefits and whole-body physiological costs. While task-dependent performance differences between static and dynamic conditions have been reported in both passive and active exoskeleton literature [21], the present study extends these observations to the QDD actuation paradigm and, critically, provides simultaneous metabolic calorimetry, surface electromyography, and kinematic assessment within a single protocol—a combination that remains rare in the field. During static holding, the device achieved shoulder muscle reductions (Upper Trapezius: −68.2%, Anterior Deltoid: −43.6%) exceeding passive spring-loaded devices and approaching upper bounds reported in systematic reviews, reflecting active torque generation capabilities that continuously adjust across shoulder postures [8,9,22]. Postural stability improvements (32–41% reductions in variability) suggest gravitational offloading reduces neuromuscular corrections required to counteract fatigue-induced drift [23], potentially preserving cognitive resources for concurrent task demands [24]. However, these localized benefits did not translate into whole-body energy savings: metabolic cost increased 57% during static holding and 30.6% during dynamic manipulation, while movement smoothness degraded by 62%. Pronounced inter-individual variability (Anterior Deltoid: −37% to −94%; Erector Spinae: −94% to +300%) underscores challenges for “one-size-fits-all” exoskeleton design, though the homogeneous participant profile (young males without industrial experience) means that the full range of variability expected in actual worker populations remains uncharacterized. The Anterior Deltoid variability was particularly notable: inclusive analysis incorporating two extreme values (>400% increase, classified as probable artifacts) reduced the responder rate from 100% to 71%, illustrating the sensitivity of small-sample responder statistics to individual data quality. This underscores the need for robust electrode attachment protocols and pre-specified artifact rejection criteria in future studies.

The exoskeleton’s ineffectiveness during dynamic tasks stems from three interrelated mechanisms. First, inertial mismatch occurs as reflected motor inertia resists rapid shoulder accelerations, offsetting gravitational support benefits—a principle demonstrated in bipedal robotics where minimizing reflected inertia improves dynamic performance [25]. Second, kinematic constraint results from the simplified 2-DOF shoulder mechanism’s inability to replicate complex scapulohumeral rhythm [17,26], generating parasitic interaction forces requiring compensatory scapular stabilizer recruitment [19,27]. Third, control strategy mismatch occurs as constant gravitational compensation cannot accommodate rapidly fluctuating inertial torques during dynamic manipulation. The self-selected pace employed in this study introduces additional variability in metabolic cost that is not attributable to the exoskeleton itself; although post hoc cycle count comparison did not reveal significant systematic pace differences between conditions (Table 4), individual pace fluctuations may have amplified or attenuated metabolic effects in ways that cannot be fully disentangled from device-related costs. Task-adaptive control architectures enabling seamless transitions between high-torque assistance and low-impedance transparency could address the control mismatch limitation [28], while metronome-paced protocols in future studies would isolate the device’s intrinsic metabolic impact from pace-induced variability. Beyond electromagnetic QDD actuators, alternative actuation paradigms such as pneumatic artificial muscles (e.g., McKibben actuators) offer high force-to-weight ratios and inherent compliance for upper-limb wearable devices [29], though their nonlinear force–length characteristics and dependence on external pneumatic supply present different deployment constraints that merit comparative investigation.

The metabolic-muscle dissociation—wherein profound shoulder offloading (Upper Trapezius: −68%, 100% responder rate) coexists with metabolic increases (static: +57%; dynamic: +31%)—challenges assumptions that localized sEMG reductions translate into whole-body energy savings. This dissociation was verified at the individual level: within the subset of participants with simultaneously valid paired data for both Upper Trapezius sEMG and metabolic cost (n = 5), four of five demonstrated concurrent muscle offloading and metabolic increase (Table 8), confirming that this pattern is not attributable to between-subject confounding from differential data availability. Schalk et al. [26] demonstrated analogous patterns, suggesting that this represents a fundamental characteristic of current assistive technology. Three mechanisms likely contribute. First, the exoskeleton’s 5 kg mass adds gravitational loading requiring unmeasured lower-limb, core stabilizer, and potentially contralateral upper-limb muscle activation. The lean participant (case4, 60 kg) was the sole individual showing static metabolic reduction (−8%), whereas the obese participant (case2, 90 kg) exhibited highest variability, suggesting device-to-body-weight ratio critically determines net metabolic impact. Looney et al. [27] predicted 0.175–0.28 W/kg metabolic increase for 5 kg torso-mounted mass, accounting for 43–68% of the observed 0.41 W/kg static increase. However, exoskeletons impose additional kinematic constraints beyond passive load carriage. Second, the device alters body segment inertial properties and center-of-mass, necessitating corrective stabilization from unmeasured lower-limb, hip, and deep core muscles. Matur et al. [29] demonstrated analogous compensation wherein trunk loading paradoxically decreased superficial spinal muscles while increasing hip extensor activity. The rigid torso coupling may constrain natural postural sway, forcing compensatory activity in unmeasured musculature. Third, continuous small-amplitude postural adjustments during nominally “static” tasks [30,31] require accelerating both biological and mechanically coupled exoskeleton components; imperfect control anticipation necessitates additional muscular force to overcome device resistance [32].

Several methodological factors should be considered when interpreting the metabolic findings. The static task metabolic data may be influenced by incomplete steady-state attainment: although the VO_2_ coefficient of variation during the final 2 min window met the conventional <10% criterion (see Section 2.4 and Figure 5), the total 2.5 min task duration provided only approximately 30 s for metabolic kinetics to stabilize before the analysis window began. The VO_2_ on-kinetics time constant for low-intensity static work is typically 30–45 s [33], suggesting that near-steady-state but not fully stabilized metabolism was captured. If donning the exoskeleton prolongs the metabolic on-transient—due to the additional postural adjustment demands imposed by 5 kg of externally mounted mass—this could asymmetrically inflate the WE condition estimate relative to WO. Furthermore, the post hoc power analysis indicated that this study was underpowered (achieved power = 0.52) for detecting metabolic differences in the observed magnitude, meaning the metabolic cost results should be considered exploratory rather than confirmatory. The absence of female participants also limits metabolic interpretation, as the 5 kg device mass represents a proportionally larger burden for individuals with lower body mass, a demographic characteristic more prevalent among female workers [34].

These findings support Howard et al.’s [10] NIOSH position calling for rigorous comprehensive evaluation prior to industrial deployment. Whole-body metabolic assessment should be mandatory alongside localized sEMG, as devices may reduce target muscle activation while imposing overall metabolic burdens undermining fatigue reduction goals. Design priorities should emphasize aggressive weight reduction (device mass accounts for 43–68% of metabolic increases), task-adaptive control incorporating real-time activity classification via multimodal sensor streams (IMU kinematics, sEMG patterns) [35,36], and enhanced kinematic compatibility through compliant interfaces accommodating scapulohumeral rhythm [16,17,18,19,26,27]. Lightweight structural materials [22], higher-torque-density motors [11,12,13,14], or hybrid passive-active architectures [22] may enable mass reduction without sacrificing assistance capability.

Individual differences in muscle offloading and metabolic responses pose significant deployment challenges. Deployment should incorporate systematic worker screening (shoulder strength assessments, range-of-motion evaluations, familiarization trials [37]) to preferentially deploy devices to workers most likely to derive net benefits.

The task-dependent effectiveness pattern directly informs potential applications. The exoskeleton demonstrates preliminary suitability for prolonged static overhead holding (>30 s cycles, >70% static content) including ceiling installation, automotive underbody assembly, and aerospace overhead riveting, where substantial shoulder reductions (44–68%) and stability improvements suggest meaningful fatigue delay potential. Conversely, high-frequency assembly (<10 s cycles, frequent tool exchanges, large-range dynamic reaching) represents inappropriate applications where 31–57% metabolic increases and 62% movement quality degradation indicate that devices impose greater burdens than benefits. These application recommendations, however, are derived from a controlled laboratory setting with naive participants performing simplified simulated tasks; confirmation in authentic industrial environments with experienced workers is necessary before definitive deployment guidance can be issued.

Future investigations should address the following specific limitations identified in this pilot study. Additionally, each participant in the present study performed only a single trial per condition without repeated measurements, precluding assessment of within-subject test–retest reliability. This design limitation means that the observed individual-level variability cannot be decomposed into true inter-individual differences versus measurement noise. Future studies should incorporate a minimum of two to three repeated trials per condition with intraclass correlation coefficient (ICC) reporting to establish measurement consistency and strengthen the evidentiary basis for individual-level responder classification. First, larger cohorts (n ≥ 15) with mixed-sex recruitment from actual industrial populations spanning 18–60 years of age are essential to establish generalizability and to identify predictors of individual response. Second, controlled task pacing (e.g., metronome-driven cycle rates) and comprehensive workload quantification (cycle counts, angular excursion, torque demand) would eliminate pace-related metabolic variability and improve reproducibility. Third, extended static holding durations (≥5 min) with verified VO_2_ plateau criteria are needed to ensure valid metabolic steady-state comparisons. Fourth, expanded sEMG arrays covering lower-limb, hip, and deep trunk muscles—or validated musculoskeletal modeling—would capture the compensatory activation patterns hypothesized to underlie the metabolic paradox. Fifth, the 2.5 min static holding protocol may not have induced meaningful fatigue in healthy young participants; prolonged or repeated-bout designs are needed to assess whether muscle offloading translates into delayed fatigue onset. Sixth, optical motion capture systems would provide higher-resolution kinematic data, addressing the IMU resolution limitations noted in the postural stability analysis (Section 3.4). Finally, multidimensional parameter space exploration (load magnitudes, assistance levels, postures) and authentic field trials at manufacturing facilities evaluating full-shift exposures, production integration, and realistic operational constraints [7,38] are necessary to bridge the gap between laboratory-demonstrated biomechanical promise and sustained industrial adoption.

## 5. Conclusions

This pilot investigation evaluated a quasi-direct-drive active upper-limb exoskeleton in seven healthy male participants (BMI: 19.6–33.1 kg/m^2^), revealing task-dependent effectiveness with critical implications for industrial deployment. Using baseline-relative sEMG normalization to circumvent MVC testing limitations, we obtained preliminary evidence for substantial biomechanical benefits during static overhead holding. The Upper Trapezius demonstrated unanimous activation reduction (−68.2%, 6/6 participants, 100% responder rate, *p* = 0.031, Cohen’s d = 3.61), representing a very large effect that is consistent across the anthropometric range of this sample. The Anterior Deltoid showed similarly large reductions (−43.6%, d = 1.31), and postural stability improved in the majority of participants with large effect sizes. These benefits appear to exceed those reported for passive spring-loaded devices, suggesting that active QDD exoskeletons may provide superior shoulder offloading during static tasks, though direct comparative studies are needed to confirm this advantage. Whether these effects generalize to female participants, older workers, and individuals with industrial work experience remains to be established.

However, a persistent metabolic cost paradox emerged: despite profound localized muscle offloading, whole-body energy expenditure increased 31% during dynamic manipulation (*p* = 0.028, statistically significant) and 57% during static holding. These metabolic findings should be interpreted as exploratory given that the study was underpowered for metabolic comparisons (post hoc power = 0.52 for the static task effect, d = 0.98).

Within-subject paired analysis confirmed that this dissociation was observed within the same individuals (4/5 participants, Table 8) rather than arising from partially overlapping analytical subsets. Only the lightest participant (60 kg) consistently showed metabolic improvements, suggesting device mass penalty (5 kg) dominates and potentially overwhelms localized muscular savings. During dynamic tasks, the exoskeleton provided minimal muscle benefits (<20% mean changes, <70% responder rates) while degrading movement quality, indicating fundamental unsuitability for high-frequency assembly requiring rapid multi-directional movements. These findings reveal a dissociation between localized biomechanical effects and systemic physiological costs—a pattern documented across exoskeleton types in recent systematic reviews—though the specific contribution of incomplete steady-state attainment, device mass, and kinematic constraint to this dissociation requires further investigation with extended protocols and larger cohorts.

Methodologically, baseline-relative sEMG normalization proved advantageous over traditional MVC methods in this study, increasing usable sample from three to six participants, substantially enhancing effect size (d: 0.95 → 3.61), revealing unanimous responder patterns, and achieving statistical significance (*p*: 0.13 → 0.031) for identical physiological effects. This supports MVC-independent assessment for intervention studies when baseline testing reliability is uncertain, a common challenge in shoulder biomechanics research. However, this approach does not replace capacity-normalized interpretation: it quantifies proportional change but cannot indicate whether offloaded muscles were operating at high or low fractions of maximum voluntary capacity, a distinction with direct ergonomic relevance. Future studies should combine both approaches using robust MVC protocols with adequate familiarization.

These findings should be interpreted within the context of a pilot study involving young male participants without industrial experience. The absence of female participants, the limited sample size, and the controlled laboratory environment constrain direct translation to occupational deployment recommendations. Nevertheless, the large observed effect size (d = 3.61) and unanimous responder pattern (6/6) for Upper Trapezius offloading within this pilot sample provide preliminary proof-of-concept warranting scaled validation in adequately powered confirmatory studies.

These preliminary findings suggest potential relevance for work scenarios involving sustained static overhead postures, such as ceiling installation, automotive undercar fastening, or aerospace overhead riveting tasks requiring extended holding periods (>2 min). The observed unanimous shoulder offloading (100% Upper Trapezius responder rate, n = 6) within this pilot sample provides initial proof-of-concept. Conversely, the metabolic burden and movement degradation observed during dynamic tasks suggest limited suitability for assembly work involving frequent directional changes or rapid cycles (<10 s). However, given that these observations are derived from a small-scale laboratory study with young male participants without industrial experience performing simplified simulated tasks, field-based validation studies with experienced workers in authentic work environments are essential before any specific deployment recommendations can be made. Such validation studies should include the full demographic range of the target workforce, including female workers and those aged 30–55 years, and should evaluate device performance under realistic production conditions, shift-length exposures, and task variability. The critical development priority remains aggressive with mass reduction toward <3.5 kg, as our sole metabolic responder (60 kg participant) suggests that the device-to-body-weight ratio represents a tipping point where mass penalty transitions from tolerable to prohibitive—though this inference is based on a single data point and requires systematic investigation across a broader body mass range.

Despite statistical power limitations (n = 6–7) and the homogeneous participant profile (young, male, non-industrial), the observed effect size (d = 3.61) and unanimous responder pattern (6/6) for Upper Trapezius offloading within this sample provide preliminary evidence warranting scaled validation in adequately powered confirmatory studies. However, the magnitude of this effect size should be interpreted cautiously given its susceptibility to inflation in small samples. Future work should prioritize larger mixed-sex cohorts (n ≥ 15) recruited from industrial worker populations, controlled task pacing protocols, extended static holding durations with verified metabolic steady-state criteria, expanded sEMG arrays capturing lower-limb and trunk compensatory muscles, and ultimately controlled field trials in authentic manufacturing environments. Only through comprehensive validation encompassing diverse demographics, realistic task conditions, and full-shift exposures can the gap between laboratory-demonstrated biomechanical promise and sustained industrial adoption be systematically addressed.

## Figures and Tables

**Table 1:** Physical information of seven volunteers.

Case	Gender	Age	Height (cm)	Weight (kg)	BMI
1	Male	24	172	65	22
2	Male	24	165	90	33.1
3	Male	24	175	70	22.9
4	Male	24	175	60	19.6
5	Male	26	179	84	26.2
6	Male	25	162	61	23.2
7	Male	24	175	92	30
Mean ± SD	-	24.4 ± 0.8	171.9 ± 6.6	74.6 ± 13.4	25.3 ± 4.8

**Table 2:** Exoskeleton technical specifications.

Component	Key Specifications
System	Mass: 5.0 kg; DOF: 2 (shoulder flexion/extension, abduction/adduction)
	Modes: Transparent (impedance compensation)/Assistive (torque support)
Actuation	Motor: GIM8115-9 with GDS810 driver (Jiangxi Sitaiwei)
	Gear ratio: 9:1 (QDD configuration); Torque: 13.0 N·m rated, 40.0 N·m peak
Power	Battery: 22.2 V Li-Po, 2500 mAh; Runtime: 3–4 h
Linkage	5-segment compliant chain (aluminum + ball bearings); Friction: ~0.01 N·m/joint
Attachment	Padded waist belt (100 mm width, 70–110 cm adjustable) with spring steel load plates

**Table 3:** Experimental protocol summary.

Phase	Duration	Task Description	Measurements
I. Baseline	5 min	Standing rest	VO_2_, VCO_2_ (resting metabolism)
II. MVC Testing	5 min	Isometric contractions (AD, UT, ES)	sEMG peak amplitude
III. Dynamic Task	5 min	Overhead screwing (repetitive reaching /manipulation)	sEMG, VO_2_, VCO_2_, IMU
IV. Static Task	2.5 min	Weighted holding (3 kg, 90°/90° posture)	sEMG, VO_2_, VCO_2_, IMU
V. Recovery	15 min	device donning-doffing/Rest	-(washout period)

Notes: Phases I–II conducted once under WO condition; Phases III–IV repeated under both WO and WE conditions in randomized order.

**Table 4:** Individual kinematic parameters during the dynamic overhead screwing task under without-exoskeleton (WO) and with-exoskeleton (WE) conditions.

SubjectID/Condition	CycleCount	MeanCycleDuration (s)	SD_CycleDuration (s)	MeanROM (°)	SD_ROM (°)
case1/WO	10	2.74	0.682	98.3	6.74
case1/WE	12	2.75	0.46	88.4	6.75
case2/WO	9	2.83	0.728	103	1.95
case2/WE	11	2.62	0.486	86	7.35
case3/WO	11	2.83	0.619	93	5.2
case3/WE	8	3.77	1.07	70.5	2.06
case4/WO	9	3.08	0.832	81.2	12.7
case4/WE	10	2.91	0.648	69	6.92
case5/WO	11	2.69	0.557	52	6.93
case5/WE	10	3.02	1.27	77.6	4.72
case6/WO	8	3.46	1.02	40.5	5.03
case6/WE	8	3.55	1.06	73.6	5.06
case7/WO	10	3.08	0.471	77.5	6.02
case7/WE	10	3.17	1.16	82.1	10.4

Notes: Inter-condition and inter-individual axis discrepancies reflect differences in sensor orientation at the time of attachment and exoskeleton-induced alterations in the dominant plane of shoulder motion. ROM: range of motion; SD: standard deviation. Cycle count reflects complete overhead screwing cycles (grasp–reach–tighten–return) identified from IMU angular velocity periodicity. Eight participants were originally recruited. One participant was excluded due to incomplete protocol completion and does not appear in any tables or figures in this manuscript. The remaining seven participants were renumbered sequentially (case1–case7) to form the final analysis cohort presented here; all seven completed the kinematic protocol under both WO and WE conditions.

**Table 5:** Dynamic overhead screwing task outcomes.

Metric	Change (%) ^a^	Responders	Median (%)	n	Cohen’s d
Metabolic Power (W)	+30.6 ± 24.7	2/6	27.1	6 ^b^	−1.24
Anterior Deltoid (sEMG)	+45.2 ± 125.8	2/6	17.1	6 ^c^	−0.36
Upper Trapezius (sEMG)	−8.5 ± 42.3	4/6	−12.4	6 ^c^	0.2
Erector Spinae (sEMG)	−15.7 ± 38.9	4/6	−18.5	6 ^c^	0.4
Angular Velocity (°/s)	−10.5 ± 23.8	4/6	−8.9	6	0.44
RMS Jerk (°/s^3^)	+32.1 ± 85.2	3/6	12.5	6	−0.38

Notes: ^a^ Change = (WE − WO)/WO × 100%; negative = improvement; values are mean ± SD. ^b^ case7 excluded from metabolic analysis due to equipment malfunction (WE = 38 W vs. WO = 116 W, −67% change deemed physiologically implausible). ^c^ Responders: number showing beneficial change (negative percentage) out of total valid participants. Key finding: Metabolic cost increased significantly (+30.6%, d = −1.24), with minimal muscle activation benefits.

**Table 6:** Static weighted holding task outcomes (3 kg load).

Metric	Change (%) ^a^	Responders	Median (%)	n	Cohen’s d
Metabolic Power (W)	+57.2 ± 58.4	1/6	116.2	6	−0.98
Anterior Deltoid (sEMG)	−43.6 ± 33.2	5/5	−37.3	5 ^b^	1.31
Upper Trapezius (sEMG)	−68.2 ± 18.9	6/6	−70.8	6 ^b^	3.61
Erector Spinae (sEMG)	−31.5 ± 67.2	4/5	−42.8	5 ^b^	0.47
Angle SD (°)	−22.3 ± 28.9	3/4	−29.6	4	0.77
Acceleration SD (m/s^2^)	−40.3 ± 20.5	4/4	−44.9	4	1.97

Notes: ^a^ Change = mean ± SD of percentage change from baseline. ^b^ sEMG baseline-relative normalization (no MVC dependencies). Key finding: Upper trapezius unanimously reduced (−68%, 6/6 responders, d = 3.61).

**Table 7:** Sensitivity of Anterior Deltoid results to exclusion of extreme values during static overhead holding.

Analysis	n	Mean ± SD (%)	Median (%)	Responders	Cohen’s d	p (Wilcoxon)
Inclusive (all valid)	7	+86.5 ± 228.1	−36.8	5/7 (71%)	−0.38	0.94
Exclusive (±400% threshold)	5	−43.6 ± 33.2	−37.3	5/5 (100%)	1.31	0.25

Notes: Two participants (case4: +488%; case6: +336%) exceeded the ±400% exclusion threshold. Positive Cohen’s d denotes beneficial effect (activation reduction); negative d denotes detrimental effect. Both analyses yield negative median values, indicating majority benefit regardless of exclusion approach. See Section 2.4 for exclusion criteria and rationale.

**Table 8:** Within-subject paired analysis of Upper Trapezius muscle offloading and metabolic cost during static overhead holding.

Participant	Body Mass (kg)	BMI	Device/Body Mass (%)	UT sEMG Change (%)	Metabolic Change (%)	Dissociation Confirmed
case1	65	22	7.7	−48.8	106.9	Yes
case3	70	22.9	7.1	−85.5	126.5	Yes
case4	60	19.6	8.3	−44.0	−8.0	No
case5	84	26.2	6	−81.1	194.7	Yes
case6	61	23.2	8.2	−89.8	8.8	Yes

Notes: Dissociation defined as concurrent UT sEMG reduction (<0%) and metabolic power increase (>0%). Device/body mass ratio = exoskeleton mass (5.0 kg)/participant body mass × 100%. Case2 excluded due to invalid UT sEMG data (+3072%, classified as artifact; see Section 2.4). case7 was excluded due to static metabolic equipment malfunction (WE = 3.07 W, physiologically implausible). UT: Upper Trapezius.

## Data Availability

The raw data and minimal dataset necessary to support the conclusions of this article will be made available by the authors upon reasonable request, and may be provided as Supporting Information files for internal evaluation purposes.
